# Evaluation of medetomidine-tiletamine-zolazepam as a partially reversible field anesthesia combination for mesocarnivores

**DOI:** 10.1371/journal.pone.0346586

**Published:** 2026-04-07

**Authors:** Fernando Nájera, Jamie L. Palmer, Stella F. Uiterwaal, Parami Peiris, Manuel Mata-Huete, Juan Francisco Sánchez, María Jesús Palacios, Rebeca Grande-Gómez, Luis Revuelta Rueda, Esther Descalzo, Jose Jimenez, Pablo Ferreras, Sharon L. Deem

**Affiliations:** 1 Karen C. Drayer Wildlife Health Center, California Carnivores Program, School of Veterinary Medicine, University of California, Davis, California, United States of America; 2 Saint Louis Zoo Institute for Conservation Medicine, Saint Louis, Missouri, United States of America; 3 Conservation Technology & Innovation, Smithsonian Conservation Biology Institute, Front Royal, Virginia, United States of America; 4 Asistencia Técnica de la Dirección General del Medio Natural y Desarrollo Sostenible de la Junta de Comunidades de Castilla- La Mancha, Toledo, Spain; 5 Dirección General de Sostenibilidad, Junta de Extremadura, Mérida, Spain; 6 Department of Animal Physiology, Faculty of Veterinary Medicine, Complutense University of Madrid, Madrid, Spain; 7 Instituto de Investigación en Recursos Cinegéticos (IREC, CSIC-UCLM-JCCM), Ciudad Real, Spain; Universidade de Trás-os-Montes e Alto Douro: Universidade de Tras-os-Montes e Alto Douro, PORTUGAL

## Abstract

Anesthesia timing stages and physiologic variables during field anesthesia with medetomidine–tiletamine–zolazepam (MTZ), partially reversed with atipamezole, were evaluated in 142 free-living individuals from 11 mesocarnivore species, including stone marten (*Martes foina*, n = 7; European badger (*Meles meles*, n = 3); European polecat (*Mustela putorious*, n = 1), Egyptian mongoose (*Herpestes ichneumon*, n = 32), common genet (*Genetta genetta*, n = 9), northern raccoon (*Procyon lotor*, n = 29), Virginia opossum (*Didelphus virginiana*, n = 27), red fox (*Vulpes vulpes*, n = 18); coyote (*Canis latrans*, n = 5), European wildcat (*Felis silvestris*, n = 7); and bobcat (*Lynx rufus*, n = 4). Intermittent live-capture operations took place in central, western, and south-central Spain and Missouri (USA) between April 2016 and August 2024. The dose of medetomidine administered ranged from 0.038–0.055 mg/kg, while that of tiletamine-zolazepam and atipamezole ranged from 2.74–4.17 mg/kg and 0.18–0.27 mg/kg, respectively. Induction times over all species were 2–7.4 minutes, while anesthesia times ranged from 36–53.56 minutes. Reversal time of the species ranged from 3–11.72 minutes and recovery times ranged from 4–44.8 minutes. During anesthesia episodes, there were no major patterns of disturbance in rectal temperature, respiratory rate, heart rate, and pulse oximetry derived hemoglobin oxygen saturation, although we did record a steady decrease of rectal temperature and heart rate over the course of the anesthesia in most animals. Based on the results obtained, MTZ dosages described produced safe physiological states and adequate anesthetic depth that facilitated minimally invasive procedures during handling of mesocarnivores in the field.

## Introduction

Field anesthesia (i.e., field chemical immobilization) is often a necessary and valuable tool for the study of free-living species’ ecology and/or health in situations when non-invasive methods are not appropriate [1]. For example, anesthesia allows researchers to safely handle animals for telemetry device attachment, to perform physical examinations, and to collect biological samples while causing minimal stress to the individual [2]. Moreover, anesthesia is often deemed mandatory for handling species that may present an inherent risk to humans if fully awake, such as with most carnivore species.

Wildlife veterinarians and biologists face the challenge of deciding which drug combination to administer based on several factors that include the target species, the procedures planned during anesthesia, and the field conditions pre-, during and post-anesthetic event. When working with multiple species, the greatest disadvantage of injectable anesthetics is individual and species variation in drug dose and drug response [3]. Combinations of an alpha-2 agonist plus a dissociative agent (e.g., xylazine or medetomidine plus ketamine) are routinely used for the anesthesia of felids although sudden arousal may occur in some species while using medetomidine (e.g., African lion *Panthera leo*, [4,5]. These combinations are commonly avoided in bears due to the possibility of sudden recoveries [6]). Additionally, as reported in the literature, the dosage may vary by species when using the same drug combination. For example, even though Sunda clouded leopards (*Neofelis diardi*) may be safely anesthetized using 0.04 mg/kg of medetomidine plus 3 mg/kg of ketamine [7], recommendations on anesthetic regimes in the mainland clouded leopard (*Neofelis nebulosa*), include the use of similar ketamine dosages (2−3 mg/kg) but medetomidine at higher doses (0.05–0.08 mg/kg; [8]. In a similar manner, the recommended tiletamine-zolazepam (TZ) dosage for the anesthesia of bobcats (*Lynx rufus*) is 10 mg/kg, while in the sister taxon, the Canada lynx (*Lynx canadensis*), it is 5 mg/kg [5]. Thus, we believe there is benefit for establishing a fixed dosage field anesthesia protocol applicable across different mesocarnivore species.

Here we explore the drug combination tiletamine (dissociative agent), zolazepam (muscle relaxant), and medetomidine (alpha-2 agonist) which can be partially reversed with atipamezole, for the field anesthesia of mesocarnivores, based on the wide use of this combination in different species of carnivores (e.g., felids such as cheetahs, *Acinonyx jubatus*, African lions, and snow leopards, *Panthera uncia*; [9,10,11]; ursids including brown bears, *Ursus arctos*, black bears, *Ursus americanus*, or polar bears, *Ursus maritimus*; [12]; [13,14]; procyonids (i.e., raccoons, *Procyon lotor*); mephitids (i.e., stripped skunks, *Mephitis mephitis*) [15], and canids, such as red foxes (*Vulpes vulpes,* [16]). In most of these cases, the dosage varies substantially depending on the species.

In this study, we aimed to explore whether an anesthetic drug combination at a fixed dosage is suitable for field anesthesia of a wide spectrum of free-living mesocarnivores. Specifically, the aims of this study were to: i) explore the efficacy, based on adequate anesthetic times (e.g., induction time, reversal time), of a medetomidine-tiletamine-zolazepam (MTZ) combination at a fixed dosage in different mesocarnivore species; ii) assess the safety of this combination by measuring four physiological parameters (i.e., heart rate, respiratory rate, body temperature, and oxyhemoglobin saturation); and iii) compare the anesthetic times and the physiological responses of this combination between the species studied.

Mesocarnivores or mesopredators are midranking mammalian predators weighing between 1–15 kg [17,18], although they can also be defined as any midranking predator in a food web, regardless of size or taxonomy [19]. Mesocarnivores attract attention in wildlife research since they play a vital role in community health and structure [20]. We focused on mesocarnivore communities from two distinct ecoregions (Southwestern Europe and Midwestern United States). None of the individuals included in this study were anesthetized for the sole purpose of this study, but were anesthetized as part of other research projects on spatial ecology and health (see [21,22]. In this study we attempt to follow the 3Rs principles of animal research applied to wildlife: (1) replacement by non-invasive methodologies, (2) reduction by maximizing the information obtained from each captured animal, and (3) refinement by using state-of-art trapping techniques and handling procedures [23], as shown in other wildlife studies (see [1]). We selected a fixed-dose protocol to evaluate the feasibility of a field anesthesia approach applicable across multiple mesocarnivore species, acknowledging the inherent interspecific variability.

## Materials and methods

We captured free-living mesocarnivores from the families *Mustelidae* (stone marten *Martes foina*, European badger *Meles meles*; European polecat *Mustela putorious*), *Herpestidae* (Egyptian mongoose *Herpestes ichneumon*), Viverridae (common genet *Genetta genetta*), Procyonidae (northern raccoon *Procyon lotor*), *Didelphidae* (Virginia opossum *Didelphis virginiana*), *Canidae* (red fox *Vulpes vulpes*; coyote *Canis latrans*), and *Felidae* (European wildcat *Felis silvestris*; bobcat *Lynx rufus*) as part of a variety of unrelated projects in Spain (Southwestern Europe) and Missouri (Midwestern United States).

In Spain, mesocarnivores were captured in two different Autonomous Communities, Extremadura (SW Spain) and Castilla-La Mancha (Central Spain). Study sites from both Autonomous Communities corresponded to Mediterranean scrublands. In Extremadura, study sites consisted mainly of private hunting estates and protected areas, with villages in and around these sites. The altitude ranged between 346–667 meters above sea level. The landscape was a mixture of cultivated lands, open oak woodlands (“dehesa”) and scrubs. Vegetation was dominated by holm oak (*Quercus ilex*) and olive (*Olea europea*) trees, with a shrub layer of Mediterranean maqui scrubland (e.g., *Erica* spp., *Cistus* spp. and *Rosmarinus* spp.) and dense scrub (*Pistacia lentiscus*, *Quercus coccifera* and *Flueggea tinctoria*) as well as open pasture areas. Land uses included extensive agriculture (cereal crops and vineyards), livestock farming and estates managed for large and small game hunting [21]. Similarly, sites at Castilla-La Mancha consisted of private hunting estates and protected areas. Landscape was dominated by sclerophyll scrublands of *Cistus* spp., *Phillyrea angustifolia*, strawberry trees (*Arbutus unedo*) and *Erica* spp., and the tree layer was primarily cork oaks (*Quercus suber*) and holm oaks (*Quercus ilex*) and included some temporary streams dominated by brambles and rushes with some ash trees (*Fraxinus angustifolia*) and pastures [24]. The altitude ranged between 125–938 meters above sea level.

In Missouri, captures occurred at three study sites, a rural area (Washington University Tyson Research Center, TRC), a suburban area (Saint Louis Zoo WildCare Park, WCP), and an urban greenspace (Forest Park). TRC is a 809 ha. and located within the Ozark Border Natural Division. The maximum and minimum elevations are 127 and 233 m. Eighty-five percent of TRC is forested with canopy trees consisting of white oak (*Quercus alba*), red oak (*Quercus rubra*), black oak (*Quercus velutina*) and pignut hickory (*Carya glabra*). The remaining 15 percent is either open grasslands or service features [25]. WCP is a 172 ha. property that exemplifies a mosaic of bottomland habitats. It includes open grasslands (part of an old golf course) and bottomland hardwood species. WCP is surrounded on the south, west, and north sides by Spanish Lake, a human modified suburban area with a population of 18,413 (www.census.gov, retrieved August 2022). The East side of the property connects with the Columbia Bottom Conservation Area, a 17.5 km^2^ floodplain located at the confluence of the Mississippi and Missouri rivers, which includes shallow wetlands, bottomland hardwoods, prairie, and cropland.

Forest Park is a 526 ha. urban park located on the western-most part of the city of Saint Louis, Missouri. One of the nation’s largest urban parks, it consists of a mosaic of land use including a zoo, multiple museums, sporting fields, two golf courses and manicured lawns. Interspersed within this park are patches of forested areas currently undergoing restoration to original oak-hickory forest, as well as a contiguous stream/pond system and a mix of prairies and marsh habitat. A network of roads, trails, and bike paths intersect the park bordered on all four sides by buildings and urban neighborhoods. Approximately 15.5 million people visit this urban park annually.

Free-living mesocarnivore captures took place during intermittent trapping seasons between April 2016-June 2021 (Spanish captures), with the majority of captures occurring from March 2017 to September 2020, and December 2021-August 2024 (United States captures), mostly from November 2022 to March 2023. All species were captured using commercial cage traps (Tomahawk models 108 and 207, Tomahawk Live Trap Co., Tomahawk, Hazelhurst, WI, USA), Safeguard model 52824, Safeguard Products/Valco Companies, Inc., New Holland, PA, USA), camtrip cage traps (Camtrip Cages, Bartsow, California, USA) or hand-made double guillotine door cage-traps, baited with road-killed deer, rabbit, squirrel, and a mixture of attractants and lures, including bobcat, red fox and coyote urine, skunk scent, and carnivore anal glands. Red foxes and coyotes were also captured using cable restraint devices intended for the capture of these species and included Belisle foot snare (Belisle enterprises, Labelle, Canada), Collarum neck restraint (Green Mountain Inc., Lander, Wyoming, USA), and the Freemont snare (Snareshop Co. Lidderdale, Iowa, USA). All captures, handling and tagging procedures in Missouri were in accordance with the Saint Louis Zoo IACUC protocol #21–15 and wildlife collectors permits #19546, 19027, and 60958 issued by the Missouri Department of Conservation (United States captures). For the work in Spain, approval was from Castilla-La Mancha University Ethics Committee and the corresponding permit (number PR-18-01-03) was granted by the Government of Castilla-La Mancha and also approved by the General Directorate of the Environment of Extremadura and Castilla-La Mancha. This work was also performed according to the regional Iberian Lynx Recovery Plans (Spain captures) under the LIFE+ project – LIFE+10NAT/ES/570. All capture and field anesthesia were performed by authorized veterinarians in compliance with Ethical Principles in Animal Research [26].

Cage traps were located in areas where previous mesocarnivore activity had been recorded via camera-traps and were camouflaged and protected from direct sunlight and rain whenever possible. Cage traps were monitored via trap alarm system (Spartan Gocam 4G/LTE cellular trail cam, Spartan Camera, Suwanee, Georgia, USA) and/or checked daily at first light and in the middle of the day (for diurnal species) to reduce holding times. Cable restraint devices were monitored via trap alarm systems to minimize holding times and reduce the risk of injury.

If a small mesocarnivore (raccoon, opossum, stone marten, badger, European polecat, Egyptian mongoose, common genet, European wildcat) was captured in a cage trap, it was transferred to a squeeze cage of known weight. We then weighed the cage with the animal to determine actual animal weight. When the squeeze cage was not available, cage traps were weighed in advance to facilitate measurement of the individual weights. For the other species (i.e., bobcat, coyote), we estimated weight by visual examination.

Once captured, cage traps and squeeze cages were covered with a dark sheet in order to prevent visual stimuli and avoid additional stress in the individuals while the team prepared the anesthetic protocol and calculated the dosages. For animals captured by cable restraint devices, preparations took place out of sight of the individual. Individuals cage-trapped in the winter in Missouri were transported into a temperature-controlled location. For all other captures, anesthesia procedures took place at the field trapping site.

For cage-trapped animals, drugs were injected intramuscularly in the hindquarters via hand-held syringe after gently squeezing the animal with the squeeze cage or using a wood panel in a standard cage trap. Mesocarnivores captured by cable restraint devices were injected intramuscularly in the hindquarters via a blowpipe using 5 ml darts (Telinject, Dudenhofen, Germany) fitted with 1.2 × 38.1 mm (18-gauge) needles (Sterile Monoject, Telinject, Germany).

We followed the MTZ anesthesia protocol with the intention to administer intramuscularly 0.04 mg/kg of medetomidine (Medetor®, Virbac, Esplugues de Llobregat, Barcelona, Spain; Placadine^TM^, Modern Veterinary Therapeutics, LLC, Miami, Florida, USA) and 3 mg/kg of tiletamine-zolazepam (Zoletil®, Virbac, Esplugues de Llobregat, Barcelona, Spain; Telazol®, Zoetis Inc., Kalamazoo, Michigan, USA) in all individuals. To antagonize the effects of medetomidine, we used 0.2 mg/kg atipamezole (Antisedan®, Orion Corporation, Espoo Finland). We administered atipamezole intramuscularly via hand-held syringe to avoid cardiovascular side effects or hyperexcitability occasionally documented with intravenous administration [27,28].

To study the anesthesia effects of this drug combination, we recorded the following timepoints: induction time (time from drug’s injection until the animal’s head rested on the ground), anesthesia time (time between animal’s head rested on the ground until atipamezole was administered), reversal time (time from atipamezole administration until the animal was showing initial signs of recovery such as ear twitching, head movement), and recovery time (time when the animal showed initial signs of recovery until it was able to stand on four limbs). We also recorded the time of release. Once the animal’s head was down and it no longer displayed auricular reflex when stimulated with a stick, we placed the animal in lateral recumbency to obtain biological samples (all species) and to fit a telemetry collar or ear tag (European wildcats, Egyptian mongooses, common genets, stone martens, red foxes, coyotes, and bobcats). For those animals that did not receive a telemetry collar or ear-tag, we clipped hair on the lateral thorax/abdomen area to aid identification in the weeks post capture and to avoid a second anesthesia. Some animals (European wildcat) could be identified by coat pattern. While processing animals in the field, we placed the individual in a shady area and protected them from rain, wind, or any other inclement weather, such as on a heated blanket for some winter procedures in Missouri. Eye lubricant ointment (Eye lube pro®, Sentrx Animal Care, Salt Lake City, Utah, U.S.A) was applied to all animals, and individuals were subsequently blindfolded to minimize visual stimuli during anesthesia.

The following physiological parameters were measured during anesthesia as in similar research (e.g., [7,9]: respiratory rate, heart rate, rectal temperature and oxyhemoglobin saturation (SpO2). To measure the respiratory rate, we observed thoracic wall movements for 30 sec. To measure oxyhemoglobin saturation (SpO2), we used a hand-held pulse oximeter (Nellcor® Oximax N-65, Nellcor Inc., Pleasanton, CA, U.S.A.) with a veterinary probe (Vetsat, Nellcor Inc.) attached to the animal’s tongue, ear or interdigital membrane. In case oxygen support was needed during the procedures (e.g., steady decrease in SpO2, i.e., < 90% SpO2 at two or more consecutive time points), oxygen could be provided via an oxygen concentrator (Inogen Rove 6, Inogen Inc., Goleta, CA, USA). To measure heart rate, we used a stethoscope, palpation of the femoral pulse and/or a pulse-oximeter. We used a digital thermometer (DataTherm II, Geratherm Medical AG, Geratal, Germany) to measure rectal temperature. We recorded all parameters at ten-minute intervals, as presented in other studies [7,11]. Muscular relaxation was subjectively measured by the control of the muscle tone in one of the hind limbs and by resistance when opening the mouth [29]. We evaluated pain perception by withdrawal reflex during venipuncture and pinching the skin between the toes [30]. Capillary refill time was evaluated for each individual. Any lesions on the body or tooth fractures found during the medical exam were recorded. Animals were weighed, and this weight was used to calculate actual dosages, standard body measurements were taken, hair was collected for genetic and hormonal studies, blood samples, feces and urine were collected whenever possible for disease studies, and a medical exam was performed on each individual, as previously described in Deem [31] and Nájera et al. [7]. Upon completion of the medical exam and/or telemetry-collar fitting, individuals were returned to the cage (cage-trapped animals) or placed in a crate (cable-restrained individuals) and atipamezole administered. Animals were released after they were not ataxic when standing.

We used the Shapiro-Wilk test to assess the normality of our data distribution. For multiple comparisons and determining differences among carnivore species in induction, reversal and recovery times, we used a one-way analysis of variance (ANOVA). When significant differences were detected (p < 0.05), post hoc comparisons were conducted using Duncan’s multiple range test to identify which species differed significantly from each other. Species represented by three or fewer individuals (i.e., badger, European polecat) were excluded from statistical comparisons to ensure robustness of the analysis (i.e., low power, which increases the chance for Type II error [32]). We used pairwise t-tests to investigate differences in anesthesia timing stages between species from the same families. Due to low sample sizes between families, only two families, Canidae (red foxes and coyotes) and Felidae (bobcats and European wildcats) could be compared.

All statistical analyses were conducted using SPSS v26.0 (IBM Corp. Released 2019. IBM SPSS Statistics for Windows, Version 26.0. Armonk, NY: IBM Corp).

## Results

We collected data from 142 individuals: stone marten, n = 7; European badger, n = 3; European polecat, n = 1, Egyptian mongoose, n = 32, common genet, n = 9, northern raccoon, n = 29, Virginia opossum, n = 27, red fox, n = 18; coyote, n = 5, European wildcat, n = 7; and bobcat, n = 4. All individuals captured presented good body condition, as indicated by body weight and muscle mass, and were considered young adults or adults based on morphometrics, body weight, and tooth wear.

**Fig 1:**
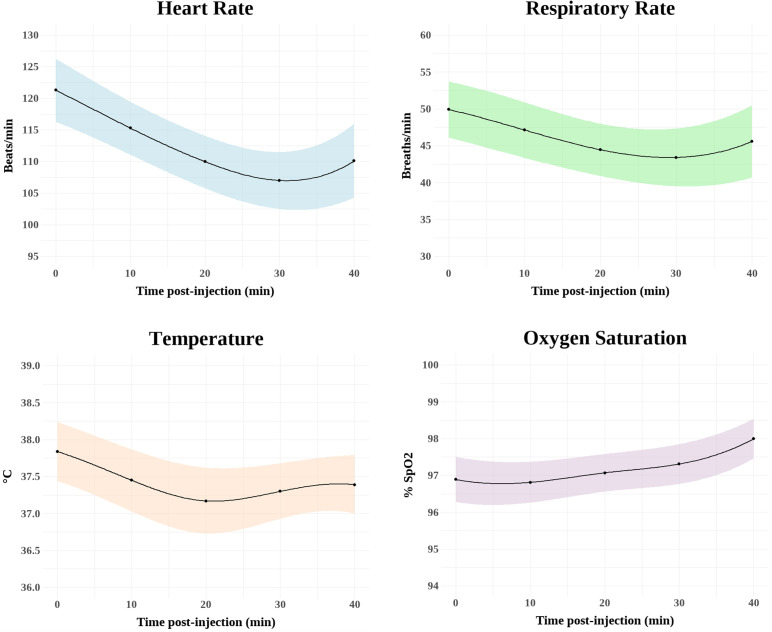
Mean heart rates, respiratory rates, SpO2 and rectal temperatures in 11 mesocarnivore species anesthetized with medetomidine-tiletamine-zolazepam. Shaded error bar denotes ± SD. Due to the normal low temperature showed during anesthetic procedures, opossums are excluded from this figure.

The mean dose of medetomidine administered ranged from 0.038–0.055 mg/kg, while that of TZ and atipamezole ranged from 2.74–4.17 mg/kg and 0.18–0.27 mg/kg, respectively. One-way ANOVA revealed minimal statistically significant differences among the nine species for the anesthetic parameters (Tables 1 and 2, and Fig 1), further described below by species. The mean induction time over all species was 2–7.4 minutes, while the mean anesthesia time ranged from 36–53.56 minutes. Mean reversal time of the species ranged from 3–11.72 min and mean recovery time ranged from 4–44.8 minutes. All animals were released back to the site of capture within 6 hours from time of first signs of recovery. Capillary refill time was < 2 sec for each animal during the length of the procedure. Muscular relaxation was considered good in all anesthesia events based on the lack of both muscular tone in the hind limbs and resistance while opening the mouth.

**Table 1:** Anesthetic parameters recorded during the field anesthesia of mesocarnivores in Spain and USA.

Species (n)	Weight (kg)	Medetomidine dose (mg/kg) [mean±sd]	TZ dose (mg/kg) [mean±sd]	Atipamezole dose (mg/kg) [mean±sd]	Induction time (min) [mean±sd]	Anesthesia time (min) [mean±sd]	Reversal time (min) [mean±sd]	Recovery time (min) [mean±sd]
**European badger (3)**	6.48 ± 3.59	0.038 ± 0.004	2.74 ± 0.45	0.187 ± 0.023	10.0 ± 4.36	36.0 ± 10.39	5.67 ± 4.04	7.67 ± 4.04
**Bobcat (4)**	8.99 ± 3.36	0.046 ± 0.01	3.47 ± 0.80	0.228 ± 0.051	4.25 ± 1.50	48.75 ± 9.64	11.50 ± 5.26*	23.50 ± 10.66
**Coyote (5)**	13.79 ± 1.78	0.055 ± 0.009	4.18 ± 0.73	0.276 ± 0.047	7.40 ± 4.34*	48.0 ± 10	7.2 ± 4.92	44.8 ± 12.85**
**Egyptian mongoose (32)**	2.27 ± 0.73	0.040 ± 0.003	3.12 ± 0.30	0.201 ± 0.010	3.97 ± 1.60	49.44 ± 6.77	7.13 ± 4.63	10.53 ± 9.89
**Red fox (18)**	4.52 ± 1.06	0.041 ± 0.004	3.10 ± 0.26	0.207 ± 0.019	4.00 ± 1.82	43.77 ± 10.15	4.21 ± 2.29	10.19 ± 9.38
**Genet (9)**	1.57 ± 0.37	0.041 ± 0.002	3.09 ± 0.21	0.202 ± 0.008	4.33 ± 0.71	53.56 ± 6.25*	10.00 ± 5.77	7.33 ± 4.74
**Opossum (27)**	2.41 ± 0.77	0.043 ± 0.007	3.28 ± 0.54	0.216 ± 0.035	3.63 ± 0.74	38.33 ± 8.11	5.37 ± 3.87	39.47 ± 43.57*
**European polecat (1)**	0.85	0.040	3.00	0.200	2.00	47.00	3.00	10.00
**Raccoon (29)**	6.24 ± 1.11	0.041 ± 0.004	3.09 ± 0.34	0.202 ± 0.023	3.66 ± 1.23	37.48 ± 5.51	11.72 ± 8.97*	28.41 ± 27.15
**Stone marten (7)**	1.25 ± 0.16	0.041 ± 0.002	3.06 ± 0.16	0.201 ± 0.012	4.14 ± 2.12	38.14 ± 13.17	5.00 ± 3.27	5.57 ± 4.20
**European wildcat (7)**	3.10 ± 0.93	0.039 ± 0.001	2.94 ± 0.10	0.194 ± 0.010	2.86 ± 0.69	49.29 ± 7.16	4.29 ± 3.04	4.00 ± 1.63

* Significant difference across all the species (p < 0.05).

** Significant difference (coyotes versus red foxes; p < 0.05).

**Table 2:** Physiological Parameters recorded during the field anesthesia of mesocarnivores in Spain and USA.

Species (n)	Physiological Parameter							
	Heart Rate (beats per minute)		Respiratory Rate (breaths per minute)		Rectal Temperature (°C)		Oxygen Saturation (%)	
	Mean±sd	Range	Mean±sd	Range	Mean±sd	Range	Mean±sd	Range
**European Badger (3)**	96.38 ± 5.96	84-104	30.67 ± 4.85	22-36	39.64 ± 0.42	39.0-40.2	95.80 ± 2.14	92-98
**Bobcat (4)**	109.0 ± 12.84	84-127	26.80 ± 7.17	18-48	38.05 ± 0.62	37.05-39.48	98.25 ± 2.12	92-100
**Coyote (5)**	87.63 ± 19.05	52-118	25.33 ± 5.87	16-36	38.31 ± 2.06	35.24-42.06	97.18 ± 2.23	92-100
**Egyptian Mongoose (32)**	115.65 ± 19.72	66-160	68.61 ± 20.7	28-120	37.50 ± 1.26	27.30-39.70	98.30 ± 2.55	89-100
**Red Fox (18)**	113.72 ± 26.95	76-220	44.81 ± 13.77	24-90	38.86 ± 0.85	36.38-40.30	97.35 ± 2.63	90-100
**Genet (9)**	146.20 ± 28.81	100-220	36.78 ± 9.00	24-56	38.81 ± 0.81	36.5-40.02	97.51 ± 3.05	89-100
**Virginia Opossum (27)**	117.99 ± 19.61	79-180	48.51 ± 10.99	30-72	33.13 ± 1.71	27-35.97	96.05 ± 3.49	85-100
**European Polecat (1)**	160.67 ± 21.68	130-176	68.00 ± 20	48-88	38.17 ± 0.54	37.6-38.9	100.00	
**Northern Raccoon (29)**	92.88 ± 19.59	53-150	30.23 ± 10.21	12-60	38.80 ± 0.54	37.23-40.1	95.78 ± 3.08	87-100
**Stone Marten (7)**	143.39 ± 20.13	103-188	47.78 ± 17.02	28-80	38.15 ± 0.77	36.6-39.7	97.12 ± 2.58	91-100
**European Wildcat (7)**	107.27 ± 15.94	80-148	25.03 ± 11.17	6-44	38.19 ± 0.65	36.6-39.4	97.57 ± 2.14	91-100
Range Values*	80-120^a^ 100-140^b^ 200-400^c^ 90-120^e^ 94-134^f^		15-30^a^ 20-30^b^ 33-36^c^		37.5-39.2^a^ 37.8-39.5^b^ 37.8-40^c^ 37.8-39.4^d^ 38.0−38.4^e^ 33.3- 35.6^g^		≥95**	

*Range values found in the literature (dog^a^, cat^b^, ferret^c^, coyote^d^, red fox^e^, northern raccoon^f^, Virginia opossum^g^) [33], [34]; [35]; [36]; [37,38,39].

** Values below 95% are considered hypoxemia [40].

While there are no normal reference vital sign values for most of the species in this study, we used range values for heart rates, respiratory rates, and rectal temperatures from domestic carnivores and those wildlife species found in the literature to determine if major disturbances occurred during the anesthetic procedures. Vital signs for all species fell within the normal reference values for domestic dogs (*Canis lupus familiaris*, 80–120 beats per minute; 15–30 breaths per minute; 37.5–39.2°C, respectively), cats (*Felis catus*, 100–140 beats per minute; 20–30 breaths per minute; 37.8–39.5°C, respectively), or ferrets (*Mustela putorious furo*, 200–400 beats per minute; 33–36 breaths per minute; 37.8–40°C) [35,39]. Coyote and red fox temperatures fell within normal temperature ranges for their species (37.8–39.4°C; [36]); raccoon temperatures and heart rates were similar to those described in the literature (38.1°C; 94–134 beats per minute, respectively, [37]. Mean rectal temperature for opossums was also similar to those previously described (33.3–35.6 °C; [38] (Fig 1; Table 2).

All the European badgers (n = 3) required additional drugs to achieve complete immobilization. Ketamine (5 mg/kg) was administered 15 minutes after initial injection of MTZ. In the rest of the species, a single MTZ administration provided adequate anesthesia depth for handling the individuals.

### Bobcat

All bobcats were radio-collared and we did not record any mortalities associated with the capture/anesthesia event in the following three months for three of the bobcats, and for one month in the fourth bobcat as the collar malfunctioned by day 30 post-deployment. This individual was camera-trapped subsequently for the following two months.

### Coyote

Coyotes were the largest species in the study group (mean weight: 13.78 kg) and exhibited a significantly longer induction time (mean = 7.40 ± 4.34 min) compared to all other species (p < 0.01). They also had the longest recovery time (mean = 44.80 ± 12.85 min), although this difference was not statistically significant. Three coyotes were captured with leg-hold snares, and two were cage-trapped. Two out of the five coyotes presented with severe full body sarcoptic mange (*Sarcoptes scabiei*) infestation. All coyotes were radio-collared and we did not detect any mortality associated with the capture/anesthesia event during three months following the procedure. One of the coyotes with severe mange died due to a *Dirofilaria immitis* infection at three months and seven days post anesthesia.

Coyotes had some of the lowest heart rate values across all time points. Similarly, they exhibited low respiratory rate values with their respiratory rate at 20, 30, and 40 minutes post-anesthetic administration, showing the lowest values across all species.

### Egyptian mongoose

We radio-collared 12.5% (4/32) of the Egyptian mongooses in this study and in these 4 animals no mortalities were recorded as a consequence of the capture (i.e., in the three months after capture). We recaptured (via camera- or cage trap) 39% (11/28) individuals up to two weeks post-capture, identified by the distinctive hair clipped pattern.

### Red fox

We radio-collared one individual and ear-tagged the other eight individuals for camera capture-recapture purposes. One red fox died a month after capture with clinical signs of a *S. scabiei* infestation. The rest of the tagged foxes were recaptured on the camera-traps between 3–12 months after the capture event. Red foxes had the shortest reversal time, although this difference was not statistically significant. Foxes generally exhibited rectal temperatures on the higher end for all time points, with a mean value of 38.86°C.

### Genet

Three out of nine genets were radio-collared and we did not detect mortalities in these individuals in the three months post-capture. Of the six genets that were identified by clipped hair, we recaptured three individuals via camera-traps for 7 days after capture. Genets had the longest anesthesia duration, were in the higher ranges for induction and reversal times, and were in the lowest ranges for recovery time.

Genets consistently exhibited the highest heart rate values (100–220).

### Virginia opossum

None of the opossums were tagged in the study. However, 44% (12/27) were recaptured and released without anesthesia during the following 5–7 nights. The 27 opossums did not show any significant patterns in the physiological parameters of heart rate, respiratory rate and oxygen saturation, although their respiratory rates were consistently on the higher end. In contrast, this species displayed the lowest rectal temperatures at all timepoints.

### Raccoon

We did not collar any of the raccoons in the study. However, 34% (10/29) were recaptured and released without a second anesthesia during the next 5–7 following nights. Although not significantly different, they had the lowest anesthetic time.. For reversal time, raccoons were one of two species (along with bobcats) with significantly longer reversal times.

Raccoons had a mean heart rate ranging from 53–150 beats per minute and were some of the lowest across all time points. Their oxygen saturation followed a similar pattern, with values constantly at the lowest of the range. Oxygen saturation was not measured at 40 minutes for this species due to the short anesthesia time in this species (37.48 ± 5.51 min). For rectal temperature, raccoons generally exhibited temperatures on the higher end.

### Stone marten

One of the stone martens was radio-collared in the study, and no mortality occurred during the three months post-capture. Two stone martens which were not collared were detected on camera-traps in the week following the capture.

### European wildcat

Two of the European wildcats were radio-collared and followed during the 6–12 months after the capture event. The rest of the individuals, identified by the pattern of the coat, were camera trapped between one week and one month after the capture. The European wildcat recorded the shortest induction time across all species. The European wildcat also had the lowest recovery time across species.

### Family specific trends

Within the Felidae family, bobcats exhibited higher recovery times compared to the European wildcat, although differences were not statistically significant. There were no other differences exhibited between the two species. Within the Canidae family, coyotes exhibited a significantly higher recovery time in comparison to red foxes (p < 0.01).

## Discussion

To the authors’ knowledge, this study is the first to present data on the use of MTZ anesthesia in eight of the study species: Egyptian mongoose, stone marten, common genet, European polecat, European wildcat, Virginia opossum, bobcat, and coyote. Although our results support the use of the MTZ combination across all 11 species studied, it is important to note that the sample size of two species, European polecat (n = 1) and European badger (n = 3), were very small. These limited sample sizes reduce the statistical power and generalizability of the findings for these species. In the case of the European badgers, we acknowledge that the additional drugs required to achieve complete immobilization may also be due to the lower dosages of medetomidine and TZ used, compared to published studies where higher doses of both drugs were described to produce adequate anesthesia (e.g., 9 mg/kg of TZ, [41]; 0.08 mg/kg of medetomidine, [42]). In our study, the results for these taxa should be interpreted with caution, and further studies with larger sample sizes are necessary to validate the efficacy and safety of MTZ anesthesia in these species. Overall, we achieved our goal of producing a fixed dose balanced/multimodal anesthetic field protocol across taxa, with lower doses for each of the drugs used to minimize adverse effects such the cardiovascular depression observed while using α2 adrenoceptor agonists drugs, and to promote hemodynamic stability [3].

Induction was considered smooth in all species, and we were able to safely handle the individuals by ten minutes after drug administration. Coyotes experienced the longest induction times compared to the rest of the species. This could be explained by the use of leg-hold snares, which we used to capture three of the five coyotes in this study. Leg-hold snares prevented us from decreasing visual stimuli while approaching the animals prior to the immobilization, potentially producing an agitated state in the individuals. In most of the species, cage traps were completely covered with a shaded cloth, helping trapped animals remain calm while we weighed the cages and calculated drug doses. Excited, aggressive, or agitated behavior has been known to affect drug absorption and result in failure to achieve optimum sedation [28,43]. Coyotes also experienced the longest recovery times. In canids, the use of tiletamine-zolazepam, although effective, often results in variable and prolonged recovery times [44,45]. Nevertheless, the addition of medetomidine and the lower doses of tiletamine-zolazepam used in this study shortened recovery times when compared to those reported when tiletamine-zolazepam was used alone [46]. Because capture methods in this study varied among species and individuals, including cage traps and cable restraint devices, future studies should consider standardizing capture methods or including capture technique as a covariate in statistical analyses to better isolate the effects of the anesthetic protocol.

In this study we aimed to administer the MTZ combination at the same dosages for all species, but overestimation of the weight in coyotes and, to a lesser extent, bobcats resulted in administering higher doses than was our target. This occurred due to the weight estimation method for these species. While in smaller carnivores, weight was calculated by subtracting the cage-trap weight from the total weight of the cage and carnivore inside the trap to yield accurate estimations, coyote and bobcat weight was assessed visually prior to anesthesia. Despite higher dosages, we did not record significant differences in anesthetic timing stages or physiological parameters. We recorded lower body temperatures in two coyotes, suggestive of hypothermia. Rather than being solely an effect of the anesthesia, the lower temperatures could also be explained by an underlying cause, since both of these individuals suffered from an advanced stage of sarcoptic mange. Sarcoptic mange is linked to reduced thermoregulation from alopecia and dysfunctional integument characteristics [47] which could have an additive effect during the anesthesia. This could also explain the longer recovery times in those individuals. Despite this finding, collar data did not suggest any obviously abnormal movements in the time they were tracked post-capture.

Lower body temperatures were consistently recorded in the Virginia opossum. However, this species is a marsupial known to maintain a body temperature of 33.3–35.6 °C [38] and mean temperatures were within that range during the procedure. Three opossums had three hypoxic oxygen saturation measurements that were resolved without supplemental oxygen and remained stable ≥95% afterwards. In this species, TZ and medetomidine-butorphanol-ketamine have been used for field immobilization [48]. TZ induction times reported were similar to those observed with MTZ.

An MTZ combination has also been used in raccoons [15], although the dosages reported differed from the ones used in this study. While Brown and Jamison [15] used a higher dose of medetomidine (0.07 mg/kg) we halved the administered dosage, attempting to decrease the adverse cardiovascular effects reported with this drug [5]. The higher dosage of TZ used in this study compared to that reported in Brown and Jamison [15] (1.7 mg/kg), may explain the shorter induction times experienced in our study. We did not find differences in reversal and recovery times between both studies. We recorded a higher heart rate, which could be due to the lower medetomidine dose employed here. We registered three partial oxygen saturation measures under 90% (89, 89, and 87%) in three individual raccoons that resolved spontaneously (i.e., in less than five minutes) without the need of oxygen support. Two of these occurred at the beginning of the procedure and we observed an upward trend during the rest of the procedure. We hypothesized that peripheral vasoconstriction or hypotension induced by medetomidine may affect the normal functioning of pulse oximeters [7].

A similar MTZ dosage has been used for the immobilization of red foxes in captivity (i.e., farmed foxes, [16]). Despite the difference in origin (captive versus free-living) anesthetic timing stages reported for captive foxes were remarkably similar to our wild-caught foxes. We expected to have different induction times since higher effective doses are often needed for free-living species over those used in captive individuals of the same species, as captive animals tend to be more docile and often more familiarized to human activity and handling [5]. We recorded heart rates within the range of resting heart rates of adult red foxes (90–120 beats minute, [33]), which was also the case for the farmed foxes. As observed in captive foxes, we noted a decrease in heart rate over time, which mainly reflects the baroreceptor reflex response to alpha-2B receptor-mediated vasoconstriction [49]. The mean respiratory rate observed in anesthetized red foxes fell within ranges reported in the literature of different species of foxes anesthetized with medetomidine-ketamine, MTZ, TZ, or xylaxine-ketamine (e.g., red fox, [16,41]; arctic fox (*Vulpes lagopus*), [27,50]; swift fox, [51]). Compared to the baseline rectal temperatures reported for red foxes (38.0–38.4 °C; [34], the rectal temperatures in our study animals were slightly higher, possibly due to the agitated state caused by the capture event [16,34], despite our efforts of decreasing the foxes’ visual stimuli once captured. We also observed a trend of decreasing rectal temperature, as recorded in farmed foxes anesthetized with MTZ [16].

TZ at 6 mg/kg has been used for field immobilization in bobcats [52], yielding longer induction and shorter anesthetic times than those reported here. Romero-Figueroa et al. [52], also reported higher heart rates. The lower heart rates recorded here may be an effect of the addition of medetomidine, which diminishes sympathetic tone and the baroreceptor reflex which may result in bradycardia [28]. A decrease in heart rates has also been recorded in domestic cats after medetomidine administration (Vahe- [53]). Heart rates in our study were similar to those described in bobcats immobilized with ketamine-medetomidine-butorphanol [54] but higher than those recorded while using butorphanol-azaperone-medetomidine [55]. The difference with the latter may be due to the higher dosage of medetomidine used (0.32 mg/kg versus 0.04 mg/kg of our study). Mean respiratory rates and rectal temperature were similar to those reported with the use of ketamine-medetomidine-butorphanol, ketamine-xylazine, and butorphanol-azaperone-medetomidine [54,55], but respiratory rates were lower than those described for the use of TZ [52]. Our protocol allowed complete immobilization with just a single administration. This differs from protocols using butorphanol-azaperone-medetomidine, where 50% of the individuals required additional drugs to allow adequate anesthesia for processing or ketamine-xylazine, and 36% of the individuals required supplementation to complete chemical restraint [55].

Egyptian mongooses and stone martens have been successfully anesthetized with α2 adrenoceptor agonists and cyclohexane combinations (i.e., xylazine-ketamine, [56]; medetomidine-ketamine, [57,1], although their research scope did not include the timing stages and/or the physiological evaluation of the combinations during the anesthetic procedures. Based on our results, our protocol provides fast induction and recovery times for the field immobilization of the species with adequate physiological parameters. [56] reported anesthetic timing stages on two individuals, and induction times (2–4 min) were similar to those described here. We detected a consistent temperature decrease during the length of the procedure. The use of TZ may cause hypothermia, which can be exacerbated when used in susceptible species, such as those with small body surface area, or in low ambient temperatures [35]. This finding was also observed in common genets. Due to the small size of these species, temperature should be monitored carefully for the duration of the procedure. Genets anesthetized with ketamine-xylazine experienced a decrease in the rectal temperature only when an additional dose was required to achieve complete immobilization, and temperatures were higher (>40°C) when capture took place in the summer [58].

The use of medetomidine/xylazine and ketamine and medetomidine-midazolam-ketamine has been used to anesthetize free-living European wildcats [59]; [60]; [61,62], although references to anesthesia timing stages remain scarce. Potoçnik et al. [59] reported longer recovery times than those described in this study, mainly due to none or a partial dose of atipamezole used in most of their immobilizations. Heart rates reported here are similar to those described in the literature (ranges: 75–134 beats per min; [62]; 76–170 beats/min, [61]). We also experienced a mild drop in heart rate during the length of the anesthesia as reported previously [61]. Respiratory rates in our study (range: 6–44 breaths/min) differed in the lower end of the range to those described by Fischer et al. [61] (20–52 breaths/min) and Bertos et al. [62] (20–80 breaths/minute). We observed an increase in respiratory rate in those wildcats (n = 2) that initially experienced <10 breaths/minute during the first period of the anesthesia (<20 minutes). Those wildcats did not receive respiratory support as the arterial oxygen saturation was ≥ 95% during the whole procedure, cyanosis was not observed in mucous membranes, and capillary refill times were <2 seconds at all time points. Rectal temperatures were within the ranges reported in other European wildcat studies [61,62]. As observed previously, rectal temperatures decreased during the course of the anesthesia. However, we only recorded one case where the final temperature dropped to 36.6°C, which is within hypothermia values (38.49 to 36.50°C) described by Redondo et al. [63] in post-anesthetized domestic cats. Moderate hypothermia has been described in European wildcats with the use of xylazine-ketamine or medetomidine-midazolam-ketamine [61,62].

Here, we demonstrate the effectiveness (based on adequate duration of the anesthetic timing stages), and physiological safety of MTZ for field anesthesia in 11 mesocarnivore species. However, we recommend further studies to identify potential physiological derangements, as many of the species we studied had low sample sizes. In this study, no other cardiovascular variables apart from heart rate and oxygen saturation were assessed to improve evaluation of the cardiovascular status. MTZ has caused hypertension and skipped heart beats in African lions, which were identified by measuring the intra-arterial blood pressure and heart rhythm [64]. Future research should determine potential negative cardiovascular effects of this combination on these species by also monitoring blood pressure, which may provide early diagnose for a wide variety of cardiovascular complications and may improve the outcome of anesthesia [65,66].

In conclusion, the MTZ dosages described here produced an adequate level of anesthesia depth that facilitated all the minimally invasive procedures needed when handling a variety of mesocarnivores in the field by providing a safe physiological response during the anesthetic event, and no post-anesthetic complications.
